# Antiprotozoal drug exposure and reduced long-term mortality and morbidity: a multi-cohort pharmacoepidemiologic study

**DOI:** 10.3389/fphar.2026.1861376

**Published:** 2026-10-27

**Authors:** Ariel Israel, Abraham Weizman, Sarah Israel, Shai Ashkenazi, Eytan Ruppin, Shlomo Vinker, Eli Magen, Eugene Merzon

**Affiliations:** 1 Department of Epidemiology and Preventive Medicine, School of Public Health, Gray Faculty of Medical and Health Sciences, Tel Aviv University, Tel Aviv, Israel; 2 Leumit Research Institute, Leumit Health Services, Tel Aviv, Israel; 3 Research Unit, Geha Mental Health Center, Felsenstein Medical Research Center, and Sagol School of Neurosciences, Faculty of Medical & Health Sciences, Tel Aviv University, Tel Aviv, Israel; 4 Department of Clinical Microbiology and Infectious Diseases, Hadassah-Hebrew University Medical Center, Faculty of Medicine, Hebrew University of Jerusalem, Jerusalem, Israel; 5 Adelson School of Medicine, Ariel University, Ariel, Israel; 6 Department of Infectious Diseases, Schneider Children’s Medical Center, Petah Tikva, Israel; 7 Cancer Data Science Laboratory, National Cancer Institute, National Institutes of Health, Bethesda, MD, United States; 8 Department of Family Medicine, Gray Faculty of Medical and Health Sciences, Tel Aviv University, Tel Aviv, Israel; 9 Medicine A Department, Assuta Ashdod University Medical Center, Ben Gurion University of the Negev, Ashdod, Israel

**Keywords:** aging, mortality, drug repurposing, pharmacoepidemiology, antiprotozoal agents, atovaquone-proguanil, mefloquine, Toxoplasma gondii

## Abstract

**Introduction:**

Medications may affect age-related morbidity and overall survival beyond their original indications. Systematic identification of such effects may inform drug repurposing strategies for healthy aging. We aimed to identify medications associated with longevity and reduced age-related morbidity using large-scale electronic health record data.

**Methods:**

We conducted a stepwise pharmacoepidemiologic investigation in two independent healthcare systems. Using electronic health records from a nationwide health provider, we performed an exploratory medication-wide case-control screen comparing medication exposure during a 10-year window between 14,986 individuals who died before sex-specific median life expectancy and matched controls who survived beyond their life expectancy. Two leading candidate drugs, atovaquone-proguanil and mefloquine, are antiprotozoal agents with activity against Toxoplasma gondii. Their effects on mortality and age-related morbidity were further evaluated within a historical cohort of 3,876 exposed individuals and 77,520 matched controls using Cox proportional hazards models adjusted for baseline characteristics. External validation was performed in the United States-based TriNetX network, including cohorts exposed to atovaquone-proguanil (n = 24,498), mefloquine (n = 3,400), and nirmatrelvir-ritonavir (n = 124,681), each matched to unexposed controls.

**Results:**

Atovaquone-proguanil and mefloquine, together with nirmatrelvir-ritonavir, a protease inhibitor with reported antiprotozoal effects, were associated with consistent reductions in all-cause mortality across cohorts and lower incidence of multiple age-related conditions, including dementia, diabetes mellitus, cardiovascular disease, chronic kidney disease, and selected malignancies. Consistent increases in specific conditions were also observed, including hearing loss, Sjögren’s syndrome/dry eye, and lichen planus, suggesting shared underlying mechanisms, although confounding cannot be excluded.

**Discussion:**

Findings were consistent across healthcare systems and drug classes, including in populations exposed to non-travel-associated medications. While causality cannot be established, these results support further investigation into potential links between chronic protozoal infections and aging-related diseases.

## 1 Introduction

Identifying the long-term effects of medications is a central goal of pharmacoepidemiology and drug repurposing research. Many approved drugs may exert previously unrecognized influences on human health, with potential benefits or harms extending beyond their original indications. Medications that promote healthy aging would be expected to reduce mortality and lower the incidence of chronic disease.

Electronic health records (EHRs) from large healthcare organizations offer a powerful resource for systematically evaluating the long-term effects of medications on mortality and age-related morbidity (Israel et al., 2024; Israel et al., 2021a; Israel et al., 2021b). Using EHR data from Leumit Health Services (LHS), a nationwide healthcare provider in Israel, we performed a medication-wide screen to identify drugs associated with increased longevity. This unbiased screen highlighted two antiprotozoal agents, atovaquone-proguanil and mefloquine—both primarily prescribed for malaria prophylaxis in travelers and not previously implicated in long-term survival or aging-related morbidity—together with nirmatrelvir-ritonavir (N-R), a more recently introduced medication with reported antiparasitic activity identified in a complementary secondary screen. Their emergence from this exploratory, hypothesis-generating screen motivated their targeted evaluation in the present study (see Methods, §2.3–2.4).

This finding raises the possibility that microbiome- or pathogen-related mechanisms contribute to the observed associations, consistent with emerging evidence linking chronic infections to cardiovascular (Wang T. et al., 2024), metabolic (Afzaal et al., 2022), malignant (Sun et al., 2025) and neurodegenerative (Toh et al., 2022; Zhou et al., 2024) disorders. Antiprotozoal agents may influence long-term health outcomes through elimination of specific protozoal pathogens and consequent effects on the microbiome and host immune responses, beyond their original therapeutic indications.

To validate and extend these exploratory findings, we conducted a series of confirmatory analyses in matched cohorts within LHS and in the independent US-based TriNetX network, an approach that also allowed us to evaluate whether associations were specific to travel-related antiprotozoal use or extended to non-traveler populations. The complete study design, comprising three sequential analyses—a discovery screen, a validation-exploration cohort, and external validation—is summarized in Figure 1 and detailed in the Methods.

**FIGURE 1:**
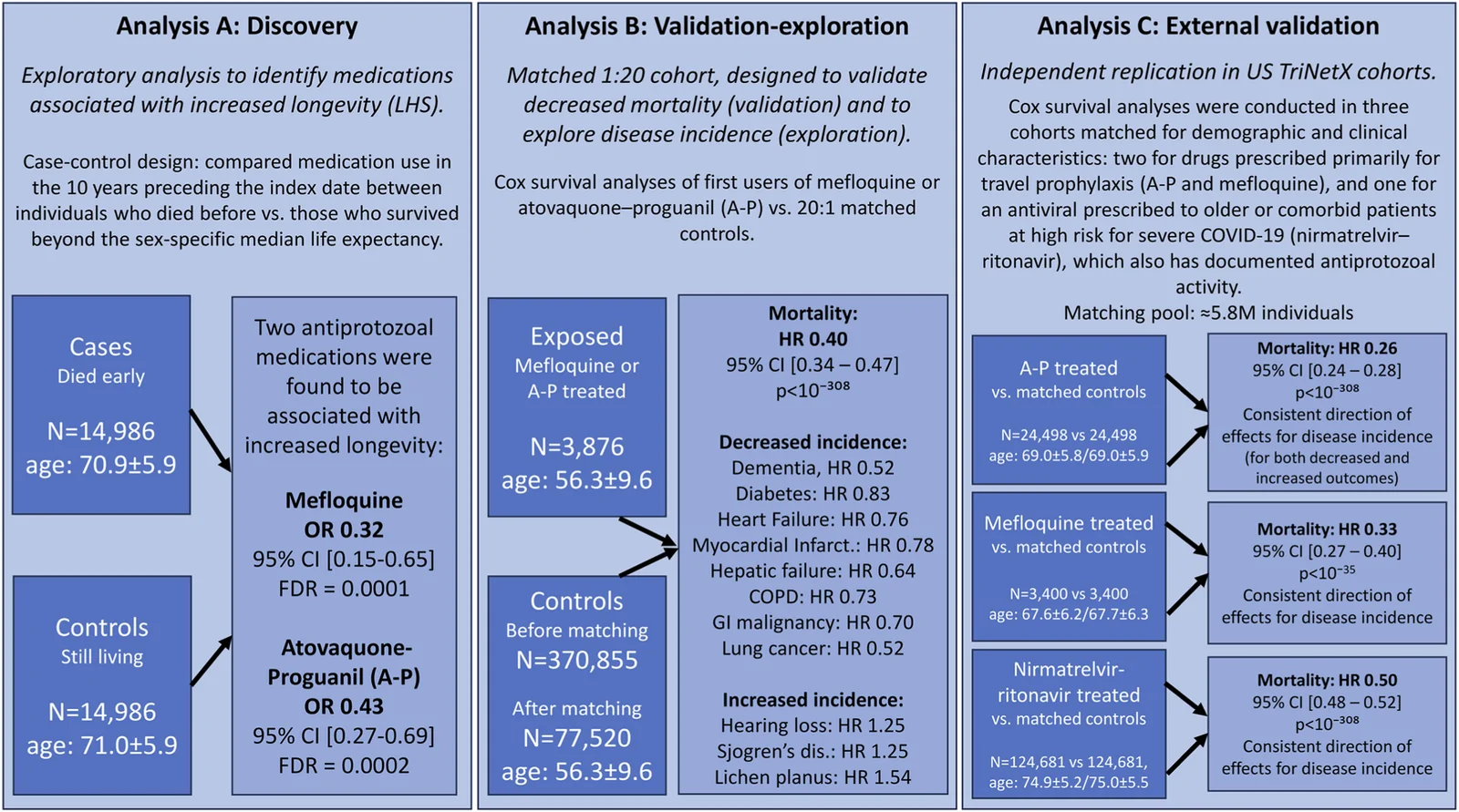
Design of the analyses performed to identify and validate medications associated with increased longevity and decreased age-related morbidity. Abbreviations: A-P: atovaquone-proguanil; CI: confidence interval; HR: hazard ratio; LHS: Leumit Health Services.

## 2 Materials and methods

### 2.1 Study design

This study was conducted in three sequential stages designed to identify and validate medications associated with increased longevity and reduced age-related morbidity (A) a medication-wide screening analysis (B) a validation and exploration cohort analysis, and (C) external replication in an independent healthcare system. The overall design is summarized in Figure 1. At each stage, strict pharmacoepidemiologic principles were applied, including alignment of time zero between exposed and unexposed individuals to avoid immortal time and related biases.

### 2.2 Data sources

#### 2.2.1 Leumit Health Services (LHS)

Leumit Health Services (LHS) is one of four nationwide healthcare providers in Israel, delivering comprehensive care to approximately 730,000 members. All Israeli citizens are entitled to universal health coverage under the national Health Basket system. LHS operates a centralized electronic health record (EHR) system with over two decades of longitudinal data, including demographics, diagnoses, laboratory results, healthcare encounters, and medication purchases. Pharmacy data include both prescription and over-the-counter medications, enabling comprehensive exposure assessment.

Data were extracted from the LHS central data warehouse through 31 December 2024, using structured query language (SQL) and Python scripts. Socioeconomic status (SES) was determined based on geocoded residential data and classified on a 1–20 scale using the Points Location Intelligence® system.

#### 2.2.2 US TriNetX network

TriNetX is a federated research network comprising electronic health records from approximately 70 healthcare organizations and more than 120 million individuals, primarily in the United States. Data include demographics, diagnoses, laboratory measurements, and medication prescriptions. Queries for this study were executed in July 2025 using the TriNetX “Compare Outcomes” analytical framework.

### 2.3 Analysis A: systematic screen procedure

The first stage (Analysis A) was a matched case-control screen designed to identify medications associated with longevity in Leumit Health Services (LHS). Eligible participants were current or former LHS members aged ≥60 years with at least 10 years of available EHR history. Individuals were required to have a minimum of 10 years of observable data following their first recorded EHR entry.

Using sex-specific longevity thresholds (84 years for females, 77 years for males), two groups were defined:

- High-longevity group: individuals alive beyond the threshold
- Below-average longevity group: individuals who died before the threshold

Each case was matched 1:1 to a control based on sex, birth year, SES group, year of first EHR record, and year of first ambulatory visit occurring at least 10 years after initial EHR entry. Matching prioritized individuals with the closest birth dates and was performed without replacement, such that each control could be matched to only one case. The index date was defined as the ambulatory visit occurring ten years after the first available EHR record for each individual, ensuring calendar adjustment and a comparable duration of electronic medical history prior to index date.

We compared, for each medication, the proportion of individuals with at least one recorded purchase in the 10 years preceding the index date. Medications were identified according to their Anatomical Therapeutic Chemical (ATC) classification, and qualifying purchases were restricted to this fixed 10-year look-back window. For each ATC class, purchase proportions were compared between the above-average and below-average longevity groups using Fisher’s exact test, with false discovery rate (FDR) correction for multiple testing. Signals were defined according to pre-specified criteria prioritizing large effect sizes (odds ratio <0.5) together with strong statistical support (FDR<0.5%), consistent with a hypothesis-generating screening objective.

### 2.4 Analysis B: validation-exploration cohort of LHS patients exposed to antiprotozoals

To validate the observed association with reduced mortality and to explore which disease reductions might underlie this effect, we constructed a cohort of individuals aged 40 to 85 who had received at least one prescription for the candidate drugs atovaquone-proguanil (A-P) and mefloquine identified in Analysis A. Each exposed individual was matched to 20 control patients of the same sex, age, socioeconomic status (SES) group, ethnic sector, and year of first EHR record, with priority given to controls with the closest birth dates. The index date was defined as the first recorded purchase of the respective medication for the case group, and this exact calendar date was assigned to each matched control. Controls were selected only from individuals confirmed to be alive and active members of LHS at that date, ensuring identical time zero for both groups and preventing immortal time and calendar biases. Matching was performed without replacement, so that each control could be used only once. Because atovaquone-proguanil and mefloquine exposures were rare (≈1% of age-eligible individuals), undocumented use prior to first EHR record would be uncommon and unlikely to materially influence exposure classification.

We compared all-cause mortality and the incidence of age-related conditions using a predefined curated list of 200 ICD-coded medical outcomes routinely monitored at Leumit Research Institute. Time-to-event analyses were conducted using Cox proportional hazards models to estimate hazard ratios (HRs) with 95% confidence intervals. Proportional hazards assumptions were assessed using Schoenfeld residuals. Additional adjustment (age, smoking status, number of ambulatory visits, pregnancy history) was applied in the Cox models, based on the patient status at their assigned index dates. Follow-up was censored at the date of death or the last recorded medical encounter for individuals who remained alive. For each outcome, individuals with a documented diagnosis prior to the index date were excluded from the corresponding analysis.

#### 2.4.1 Secondary screen for identification of non-travel associated medications with similar effects

Because atovaquone-proguanil and mefloquine are primarily prescribed for travel-related indications, we considered the possibility of travel-associated confounding. To address this, we performed a secondary exploratory screen using a shorter 5-year exposure window to identify medications with potential antiprotozoal activity that were not linked to travel-related use.

This approach identified nirmatrelvir-ritonavir (N-R, Paxlovid), a protease inhibitor introduced in 2021 and widely used for the prevention of severe outcomes in patients with COVID-19. Ritonavir has been reported to exhibit activity against intracellular protozoal pathogens.

In our health system, nirmatrelvir-ritonavir was provided free of charge to eligible patients using centrally applied criteria and distributed through a dedicated response team independent of the primary treating physician (Israel et al., 2022).

Although not captured in the 10-year screening window of Analysis A due to its recent introduction, this medication was included as an additional comparator in the validation analyses to assess whether similar associations could be observed in a population not selected by travel-related exposure.

### 2.5 Analysis C: TriNetX validation cohort

To validate the mortality reduction observed in LHS and to test whether associations could be reproduced in an independent U.S. population, we conducted analyses using TriNetX, a federated research network comprising electronic health record (EHR) data from 70 healthcare organizations and covering more than 120 million patients. Queries were executed online in July 2025.

Validation cohorts included individuals aged 70–89 years at the time of cohort extraction who had either a documented prescription or a qualifying ambulatory visit, which served as the index event. In TriNetX, current age is computed automatically as the time elapsed between the individual’s date of birth and cohort extraction date, irrespective of whether the individual is alive or deceased. For decedents, age is not derived from the date of death; the system abstracts the date of birth and applies the same extraction timestamp used for the cohort query. Accordingly, current age does not condition on survival status and cannot introduce immortal-time bias.

Eligibility additionally required documentation of BMI within the year preceding the index event, ensuring that metabolic matching was based on measurements with comparable recency in all participants. Cases were defined as individuals with a recorded prescription for the medication of interest (atovaquone–proguanil, mefloquine, or nirmatrelvir–ritonavir); controls were individuals with a qualifying ambulatory visit, both within the same BMI-referenced window.

In TriNetX, the index date is automatically defined as the first point at which all inclusion criteria are simultaneously satisfied. For exposed individuals, this necessarily corresponds to their first recorded prescription, and follow-up contributes only after this event; no person-time prior to exposure contributes to the exposed cohort. We used the TriNetX Compare Outcomes pipeline, in which exposed individuals are matched 1:1 to controls using greedy nearest-neighbor propensity-score matching without replacement, ensuring that each control is used only once. Controls were not required to remain unexposed during follow-up, thereby avoiding bias from conditioning on future exposure. TriNetX simply prevents individuals in the exposed cohort from being simultaneously selected as controls, without restricting subsequent medication use. Propensity score matching (PSM) was applied to balance groups on age, sex, ethnicity, race, smoking status, diabetes diagnosis, BMI, and HbA1c levels at baseline. For nirmatrelvir-ritonavir, availability beginning in 2021 resulted in a modest calendar-time shift, which is intrinsic to its recent introduction. Outcomes were compared using Kaplan-Meier survival curves and Cox proportional hazards models to estimate hazard ratios (HRs) with 95% confidence intervals.

Guided by the observed associations in LHS, we evaluated the following outcomes:

- All-cause mortality
- Diabetes (ICD-10: E08-E13; or incident HbA1c ≥ 6.5%)
- Dementia (F03, G30)
- Renal insufficiency (eGFR <45 mL/min/1.73 m^2^), chosen to ensure a uniform and clinically meaningful definition across healthcare organizations
- Hepatic failure (K72)
- Congestive heart failure (CHF) (I50)
- Myocardial infarction (MI) (I21)
- Malignancies: gastrointestinal (C15-C26), urinary tract (C64-C68), brain (C71), lung (C30-C39)
- Chronic Obstructive Pulmonary Disease (COPD) (J43-J44)
- Hearing loss (H90-H91)
- Sjögren’s syndrome (M35.0)
- Retinal detachment (H33)
- Lichen planus (L43)

These codes were intentionally restricted to ensure uniform diagnostic specificity across healthcare systems, acknowledging reduced sensitivity but minimizing differential misclassification bias. Follow-up was censored at the date of the last recorded medical encounter, up to 4000 days (∼11 years) after the index date. For each outcome, individuals with a documented diagnosis prior to the index date were excluded from the corresponding analysis.

### 2.6 Data processing in LHS

Data from LHS were extracted from the centralized EHR system and processed using T-SQL queries and Python 3.11 scripts developed by the Leumit Research Institute. To ensure anonymity, all patient identifiers were removed and replaced with study-specific codes prior to analysis. Medication exposure was determined from pharmacy purchase records, covering both prescription and over-the-counter drugs, up to 10 years prior to the index date. Medications were classified according to the Anatomical Therapeutic Chemical (ATC) system.

### 2.7 Statistical analyses

For LHS data, Fisher’s exact test was used to compare categorical variables. Given the very large sample sizes, t-tests were considered robust to moderate departures from normality under the central limit theorem. Survival between treatment groups was compared using the log-rank test. All tests were two-tailed, with P-values below 0.05 considered statistically significant. Analyses of LHS data were conducted in R version 4.4.

For TriNetX data, analyses were performed within the TriNetX platform using the built-in “compare outcome” analysis pipeline, which applies Kaplan-Meier estimators and Cox proportional hazards models to propensity score matched cohorts.

## 3 Results

### 3.1 Analysis A: LHS exploratory cohort

Table 1 summarizes the demographic and clinical characteristics of 14,986 patients who died before the cohort median age of death (cases) and 14,986 matched survivors (controls) in the LHS cohort. Matching ensured similar distributions of age, sex, and socioeconomic status (50.8% female, mean age 71.0 ± 5.9 years at index). Compared to controls, cases had slightly higher BMI (29.1 ± 6.0 vs. 28.5 ± 4.8 kg/m^2^) and weight (77.5 ± 17.7 vs. 76.3 ± 14.6 kg) and were more likely to be smokers (OR 2.00, 95% CI 1.86-2.15). After an average follow-up of 12.5 years, controls reached a mean age of 83.6 ± 6.5 years, while cases died at a mean age of 74.3 ± 5.6 years (Table 1B).

**TABLE 1:** Demographic and clinical characteristics of the LHS exploratory longevity cohort (Analysis A). A. at the index date.

​	​	Case N = 14,986	Control N = 14,986	P-value	Odds ratio	SMD
A. at the index date						
Sex	Female	7,617 (50.8%)	7,617 (50.8%)	1.000	1	​
	Male	7,369 (49.2%)	7,369 (49.2%)	1.000	1	​
Age (years)	​	70.9 ± 5.9	71.0 ± 5.9	0.098	​	−0.019
Age category	60–69	6,311 (42.11%)	6,244 (41.67%)	0.433	1.02 [0.97 to 1.07]	​
	70–79	7,303 (48.73%)	7,317 (48.83%)	0.881	1.00 [0.95 to 1.04]	​
	80–89	1,372 (9.16%)	1,425 (9.51%)	0.293	0.96 [0.89 to 1.04]	​
Year of index date	mean ± SD	2012 ± 2	2012 ± 2	1.000	​	0.000
	median [IQR]	2013 [2012; 2013]	2013 [2012; 2013]	​	​	0.000
Year of first EHR record	mean ± SD	2002 ± 2	2002 ± 2	1.000	​	0.000
	median [IQR]	2003 [2002; 2003]	2003 [2002; 2003]	​	​	0.000
Weight (kg)	​	77.5 ± 17.7	76.3 ± 14.6	<0.001	​	0.076
Height (cm)	​	165 ± 10	164 ± 10	0.030	​	0.030
Body Mass Index (kg/m^2^)	​	29.1 ± 6.0	28.5 ± 4.8	<0.001	​	0.127
BP systolic (mmHg)	​	134 ± 20	133 ± 17	0.104	​	0.019
BP diastolic (mmHg)	​	76.8 ± 10.1	77.5 ± 9.0	<0.001	​	−0.079
Smoking status	Non-smoker	6,705 (73.6%)	12,067 (84.3%)	<0.001	​0.52 [0.49 to 0.55]	​
	Past smoker	485 (5.32%)	562 (3.93%)	<0.001	1.38 [1.21 to 1.56]	​
	Smoker	1,919 (21.1%)	1,684 (11.8%)	<0.001	2.00 [1.86 to 2.15]	​
Socio-economic status (1-20)	​	9.50 ± 3.14	9.55 ± 3.17	0.266	​	​
B. at the end of follow-up						
Age (years) #	​	74.3 ± 5.6	83.6 ± 6.5	<0.001	​	−1.213
​	60–69	3,027 (20.2%)	​	<0.001	Inf [1050.26 to Inf]	​
​	70–79	8,542 (57.0%)	4,639 (31.0%)	<0.001	2.95 [2.81 to 3.09]	​
​	80–89	3,416 (22.8%)	7,516 (50.2%)	<0.001	0.29 [0.28 to 0.31]	​
​	90-	​	2,831 (18.9%)	<0.001	0.00 [0.00 to 0.00]	​
died during period	​	14,986 (100%)	​	​<0.001	Inf [8402.05 to Inf]	

Panel A shows baseline demographic and clinical characteristics at the index date, comparing individuals who died before reaching the sex-specific median life expectancy (cases) with matched individuals who survived beyond it (controls). Panel B shows the same characteristics at the end of follow-up.

Continuous variables are presented as mean ± standard deviation (SD), with p-values from two-tailed t-tests. Categorical variables are shown as counts and percentages, with p-values from Fisher’s exact tests. Odds ratios (OR) with 95% confidence intervals (CI) represent associations with case status. Standardized mean differences (SMD; Cohen’s d) are provided as measures of effect size.

IQR: interquartile range.

# Here, age reflects the maximal age reached at the end of follow-up, taken at the date of death for deceased individuals.

Abbreviations: SD: standard deviation; IQR: interquartile range; OR: odds ratio; CI: confidence interval; SMD: standardized mean difference; BP: blood pressure; EHR: electronic health record.

### 3.2 Analysis A results: medications associated with decreased mortality

Medication use during the preceding decade was systematically evaluated for associations with mortality. Out of 1470 screened compounds, seven agents, from diverse therapeutic classes met the prespecified OR and FDR threshold (OR<0.5, FDR <0.5%, Benjamini-Hochberg correction). The complete list of screening signals is provided in Supplementary Table S2. The ATC class P01B (antimalarials) was the only therapeutic category represented by multiple top entries, prompting downstream validation of these two antiprotozoal agents: mefloquine (OR 0.32, 95% CI 0.15–0.65, FDR = 0.0001) and atovaquone–proguanil (OR 0.43, 95% CI 0.27–0.69, FDR = 0.0002) (Supplementary Table S1).

### 3.3 Analysis B: validation-exploration cohort in LHS

These findings prompted a more detailed evaluation of mortality and morbidity outcomes in matched cohorts. We constructed a cohort of individuals aged 40-85 who had received at least one prescription for either A-P or mefloquine, and matched each treated patient to 20 unexposed controls based on sex, socioeconomic status (SES), and year of first EHR record. The final cohort included 3,876 treated individuals and 77,520 controls with broadly comparable baseline characteristics (Table 2).

**TABLE 2:** Demographic and clinical characteristics of the LHS cohort of individuals treated with atovaquone-proguanil or mefloquine versus matched controls (Analysis B).

​	​	Case N = 3,876	Control N = 77,520	P-value	Odds ratio	SMD
Sex	Female	2,028 (52.3%)	40,560 (52.3%)	1.000	1	​
	Male	1,848 (47.7%)	36,960 (47.7%)	1.000	1	​
Age at index (years)		56.3 ± 9.4	56.3 ± 9.6	0.990		
Age category	**40–49**	1,100 (28.4%)	21,624 (27.9%)	0.509	1.02 [0.95 to 1.10]	​
	**50–59**	1,325 (34.2%)	27,903 (36.0%)	0.023	0.92 [0.86 to 0.99]	​
	**60–69**	1,111 (28.7%)	20,594 (26.6%)	0.004	1.11 [1.03 to 1.19]	​
	**70–79**	326 (8.41%)	6,389 (8.24%)	0.697	1.02 [0.91 to 1.15]	​
	**80–89**	14 (0.36%)	1,010 (1.30%)	<0.001	0.27 [0.15 to 0.46]	​
Year of index date	mean ± SD	2015 ± 4	2015 ± 4	1.000	​	0.000
	median ]IQR]	2016 [2013; 2018]	2016 [2013; 2018]	​	​	0.000
Weight (kg)		76.3 ± 15.3	77.3 ± 16.5	<0.001	​	−0.060
Height (cm)		168 ± 9	167 ± 10	<0.001	​	0.079
Body Mass Index (kg/m^2^)		27.0 ± 4.5	27.5 ± 5.2	<0.001	​	−0.114
BP systolic (mmHg)		125 ± 16	127 ± 17	<0.001	​	−0.098
BP diastolic (mmHg)		76.9 ± 9.1	77.4 ± 9.6	0.004	​	−0.048
Smoking status	Non-smoker	3,168 (82.8%)	56,248 (75.3%)	<0.001	1.58 [1.45 to 1.73]	​
	Past smoker	105 (2.74%)	2,181 (2.92%)	0.587	0.94 [0.76 to 1.15]	​
	Smoker	553 (14.5%)	16,301 (21.8%)	<0.001	0.61 [0.55 to 0.66]	​
Ethnic sector	Arab	88 (2.27%)	1,760 (2.27%)	1.000	1	​
	General	3,591 (92.7%)	71,820 (92.7%)	1.000	1	​
	Jewish ultra-orthodox	197 (5.1%)	3,940 (5.1%)	1.000	1	​

Baseline demographic and clinical characteristics of Leumit Health Services (LHS) patients exposed to atovaquone-proguanil or mefloquine (cases) compared with matched controls. Matching was performed on birth year, sex, ethnic sector, year of first EHR record, and socioeconomic status (SES) category. Reported values reflect characteristics at the index date (first prescription for cases; assigned index date for controls).

Counts and percentages represent categorical variables at baseline. Odds ratios (OR) with 95% confidence intervals (CI) are shown for categorical variables, with p-values from Fisher’s exact test. Continuous variables are presented as mean ± standard deviation (SD), with standardized mean differences (SMD; Cohen’s d) provided as effect size estimates.

Abbreviations: SD, standard deviation; IQR, interquartile range; OR, odds ratio; CI, confidence interval; SMD, standardized mean difference; BP, blood pressure; BMI, body mass index.

The mean age at index date was 56.3 years in both groups, with 52.3% female participants. Small but statistically significant baseline differences were noted for BMI (27.0 ± 4.5 vs. 27.5 ± 5.2 kg/m^2^, p < 0.001), weight (76.3 ± 15.3 vs. 77.3 ± 16.5 kg, p < 0.001), and height (168 ± 9 vs. 167 ± 10 cm, p < 0.001). Systolic blood pressure (125 ± 16 vs. 127 ± 17 mmHg, p < 0.001), and diastolic blood pressure (76.9 ± 9.1 vs. 77.4 ± 9.6 mmHg, p = 0.004). Current smoking was significantly less frequent in the treated group (14.5% vs. 21.8%, p < 0.001), although past smoking rates and ethnic sector distributions were similar between cases and controls.

### 3.4 Analysis B: Cox proportional hazards models

We next evaluated the association between exposure to A-P or mefloquine and subsequent mortality and morbidity using Cox proportional hazards models (cumulative incidence plots in Figure 2). Treated individuals had markedly reduced risk of all-cause mortality (HR = 0.40 [95% CI, 0.34-0.47], p < 0.001; Figure 2A). Significant reductions were also observed for dementia (HR = 0.52 [0.41-0.67], p < 0.001; Figure 2B), diabetes mellitus (HR = 0.83 [0.75-0.92], p = 0.0006; Figure 2C), heart failure (HR = 0.76 [0.63-0.91], p = 0.003; Figure 2D), myocardial infarction (HR = 0.78 [0.66-0.92], p = 0.003; Figure 2E), hepatic failure (HR = 0.64 [0.43-0.95], p = 0.03; Figure 2F), chronic obstructive pulmonary disease (COPD) (HR = 0.73 [0.62-0.86], p = 0.0002; Figure 2G), gastrointestinal malignancy (HR = 0.70 [0.52-0.96], p = 0.03; Figure 2H), and lung cancer (HR = 0.52 [0.33-0.82], p = 0.004; Figure 2I).

**FIGURE 2:**
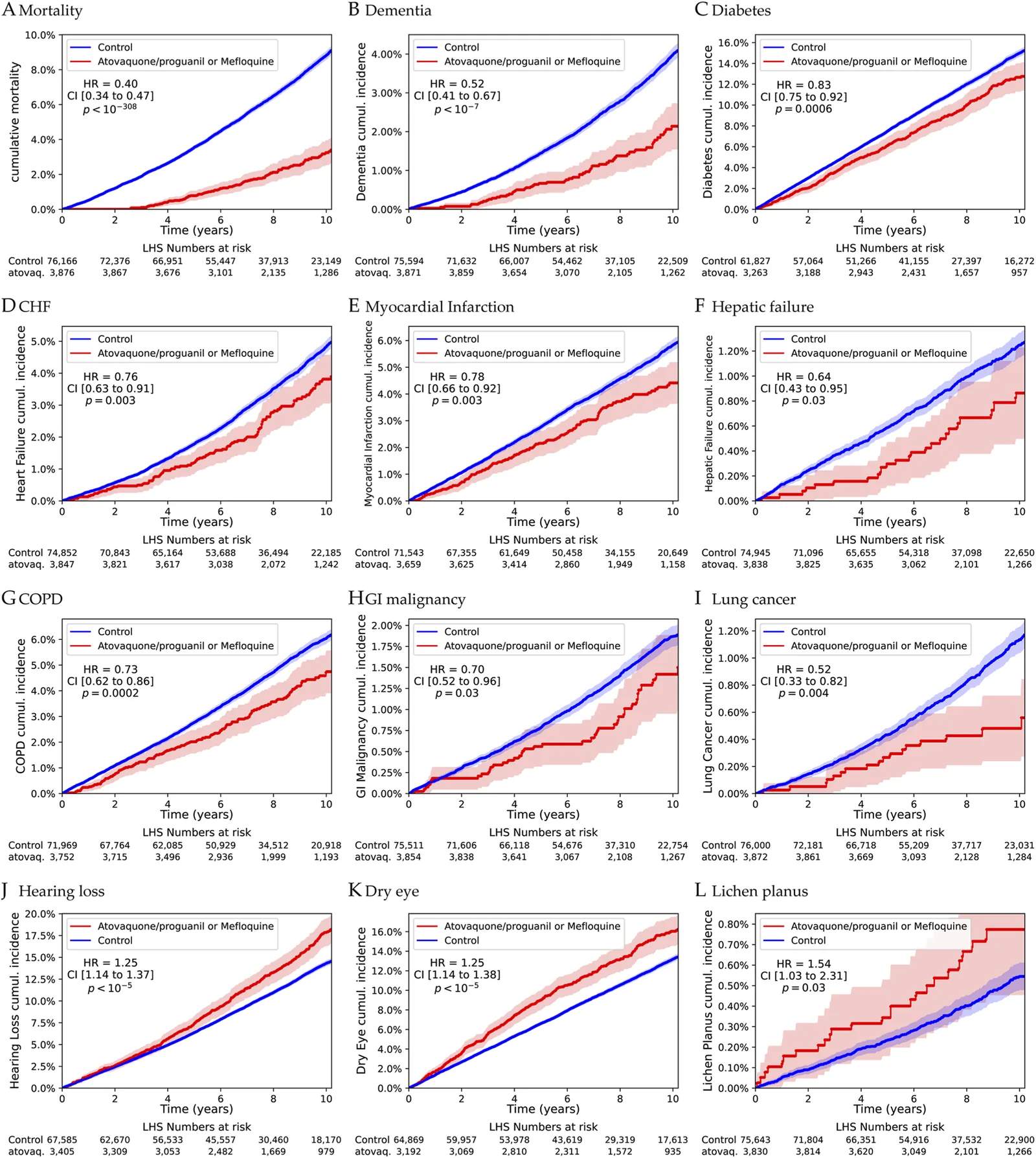
Cumulative mortality and incidence of major health outcomes in patients treated with antiprotozoals versus matched controls in LHS (Analysis B). Kaplan-Meier curves showing cumulative incidence of major health outcomes among Leumit Health Services (LHS) patients exposed to mefloquine or atovaquone-proguanil (red) compared with matched controls (blue). Hazard ratios (HR), 95% confidence intervals (CI), and p-values were estimated from Cox proportional hazards models. Follow-up was censored at the date of death or last recorded visit, whichever occurred first, and individuals with a diagnosis of the outcome prior to the index date were excluded. Shaded areas represent 95% CI. Panel labels: **(A)**, all-cause mortality; **(B)**, dementia; **(C)**, diabetes mellitus; **(D)**, congestive heart failure (CHF); **(E)**, myocardial infarction; **(F)**, hepatic failure; **(G)**, chronic obstructive pulmonary disease (COPD); **(H)**, gastrointestinal (GI) malignancy; **(I)**, lung malignancy; **(J)**, hearing loss; **(K)**, dry eye; **(L)**, lichen planus. Abbreviations: A-P: atovaquone-proguanil; CI: confidence interval; HR: hazard ratio; LHS: Leumit Health Services.

Conversely, treated individuals exhibited significantly increased risks of hearing loss (HR = 1.25 [1.14-1.37], p < 0.001; Figure 2J), Sjögren’s disease/eye dryness (HR = 1.25 [1.14-1.38], p < 0.001; Figure 2K), and lichen planus (HR = 1.54 [1.03-2.31], p = 0.03; Figure 2L).

A forest plot summarizing these outcomes (Figure 3) illustrates the broad reductions in mortality and major age-related conditions associated with antiprotozoal exposure, alongside increased risks of specific auditory, ophthalmic, and dermatologic conditions.

**FIGURE 3:**
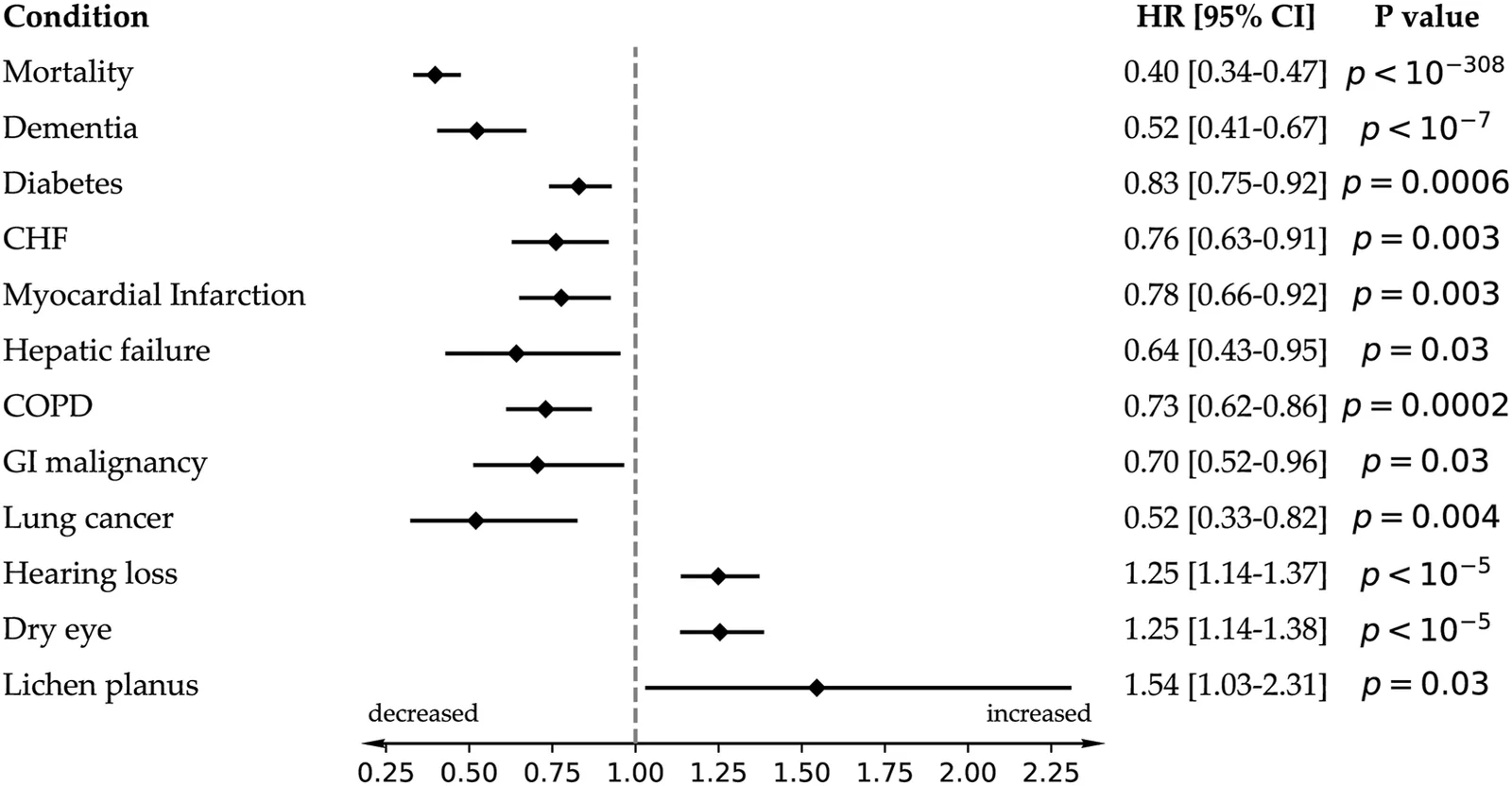
Forest plot of selected health outcomes in patients treated with atovaquone-proguanil or mefloquine vs. matched controls in LHS (Analysis B). Hazard ratios (HR) and 95% confidence intervals (CI) for all-cause mortality and selected age-related conditions among patients treated with mefloquine or atovaquone-proguanil, compared with matched controls in Leumit Health Services (LHS). Outcomes were estimated using Cox proportional hazards models, with follow-up censored at the date of death or last recorded visit, whichever occurred first. Individuals with a diagnosis of the respective condition prior to the index date were excluded. Horizontal bars indicate 95% CIs; values to the left of 1 indicate reduced risk, and those to the right indicate increased risk. Protective associations were observed for mortality, dementia, diabetes mellitus, cardiovascular disease, hepatic failure, chronic obstructive pulmonary disease, and selected malignancies. Increased risks were observed for hearing loss, dry eye, and lichen planus. Abbreviations: CHF: congestive heart failure; CI, confidence interval; HR, hazard ratio; LHS, Leumit Health Services; COPD, chronic obstructive pulmonary disease; GI, Gastrointestinal.

In addition, given that atovaquone-proguanil and mefloquine are primarily prescribed for travel-related indications, we conducted a secondary exploratory screen within LHS using a shorter 5-year exposure window to identify medications with potential antiprotozoal activity not linked to travel-related use. This analysis identified nirmatrelvir-ritonavir (N-R, Paxlovid), a protease inhibitor introduced in 2021 and widely used for the prevention of severe outcomes in patients with COVID-19. Ritonavir has been reported to exhibit activity against intracellular protozoal pathogens, providing biological plausibility for shared effects with A-P and mefloquine. Results of an exploratory matched comparison are provided in Supplementary Table S3.

Although not captured in the 10-year screening window of Analysis A due to its recent introduction, N-R was identified in the secondary LHS screen and included as an additional comparator in subsequent validation analyses to provide a non-travel-associated exposure context.

### 3.5 Analysis C: validation cohorts in the US-based TriNetX collaborative network

The TriNetX network provides an opportunity to validate the observed associations in a large, independent Western population, comprising over 120 million individuals, using standardized and widely adopted cohort construction and analysis pipelines. We used this platform to validate the associations observed in LHS for each of the medications identified in Analysis B.

We constructed large historical prospective cohorts within TriNetX. In these analyses, we included a third validation cohort exposed to nirmatrelvir-ritonavir (N-R), identified in the extended LHS screen. The clinical setting in which this medication is used - prevention of severe COVID-19 outcomes in comorbid older individuals - provides a validation context independent of travel-related use.

Moreover, owing to its widespread use during the COVID-19 pandemic, the N-R cohort provided substantial statistical power. However, its recent introduction limits follow-up duration to approximately three years, in contrast to the longer observation periods available for atovaquone-proguanil and mefloquine.

For each of the three medications, we constructed matched cohorts of individuals aged 70–89 years at the time of cohort extraction, irrespective of vital status. Cases were defined as individuals with a recorded prescription for the medication of interest, while controls were individuals without such exposure at baseline. Propensity score matching (PSM) was applied to balance groups on age, sex, race, ethnicity, smoking status, diabetes, BMI, and HbA1c at baseline.

The final matched cohorts included 24,498 exposed individuals and 24,498 controls for A-P, 3,400 exposed and 3,400 controls for mefloquine, and 124,681 exposed and 124,681 controls for N-R (Table 3). Across cohorts, mean age at index was closely matched (69.0 ± 5.8 years for A-P, 67.6 ± 6.2 years for mefloquine, and 75.0 ± 5.5 years for N-R). The close equivalence of both age at index and current age after matching empirically confirms identical follow-up time windows in exposed and unexposed groups, supporting the absence of immortal-time and calendar bias. Sex distributions were balanced (55% female for A-P, 52% for mefloquine, 58% for N-R). Race and ethnicity distributions were similar within each matched cohort, with the majority identified as White (83% for A-P, 56% for mefloquine, 85% for N-R). Baseline prevalence of nicotine dependence (3%–8%), diabetes mellitus (14%–26%), and overweight/obesity (13%–27%) were comparable between groups. HbA1c and BMI were also closely matched, ensuring well-balanced baseline metabolic profiles for subsequent outcome analyses.

**TABLE 3:** Demographic and clinical characteristics of the matched TriNetX cohorts for atovaquone-proguanil, mefloquine, and nirmatrelvir-ritonavir exposure (Analysis C).

​		Atovaquone-proguanil	Mefloquine	Nirmatrelvir-ritonavir
N		24,498/24,498	3,400/3,400	124,681/124,681
Age at Index		69.0 ± 5.8/69.0 ± 5.9	67.6 ± 6.2/67.7 ± 6.3	74.9 ± 5.2/75.0 ± 5.5
Current age*		76.7 ± 4.8 /76.8 ± 5.0	76.8 ± 5.0 /76.1 ± 4.9	77.0 ± 5.1 /80.3 ± 5.4
Sex	Female	55.0%/55.0%	52.0%/52.5%	57.7%/58.1%
	Male	45.0%/45.0%	48.0%/47.5%	42.3%/41.9%
Race/Ethnicity	White	82.8%/83.3%	55.4%/56.2%	86.7%/83.5%
	Black or African American	5.3%/5.2%	15.7%/15.4%	4.7%/4.8%
	Hispanic or Latino	1.2%/1.1%	1.3%/1.0%	2.8%/2.7%
	Not Hispanic or Latino	83.8%/79.7%	82.5%/78.0%	73.1%/75.7%
	Asian	4.5%/4.2%	13.4%/13.3%	3.4%/4.1%
	Other Race	1.6%/1.8%	4.4%/3.5%	1.9%/2.5%
Nicotine dependence		3.5%/3.3%	3.1%/2.7%	7.9%/7.5%
Diabetes mellitus		14.0%/13.6%	22.3%/21.9%	26.0%/25.4%
Overweight or obesity (diagnosis)		14.4%/14.3%	13.0%/13.4%	26.8%/25.6%
HbA1c (%)		5.9 ± 0.9/5.9 ± 1.1	6.3 ± 1.1/6.2 ± 1.2	6.0 ± 1.0/6.0 ± 1.0
Hemoglobin A1c range	0%–5.7%	25.6%/25.4%	15.9%/15.7%	35.9%/33.7%
	5.7%–6.5%	25.5%/24.9%	27.1%/26.5%	38.7%/35.2%
BMI (kg/m^2^)		26.8 ± 4.8/26.8 ± 4.9	27.4 ± 4.9/27.2 ± 5.1	28.2 ± 5.9/28.2 ± 5.8

Baseline demographic and clinical characteristics of patients exposed to atovaquone-proguanil, mefloquine, or nirmatrelvir-ritonavir and their propensity score-matched controls in the TriNetX network. Matching was performed on age, sex, race, ethnicity, smoking status, diabetes diagnosis, BMI, and HbA1c levels at baseline. Reported values reflect characteristics at the index date (first prescription for exposed patients; matched ambulatory visit for controls).

Values are presented as mean ± standard deviation (SD) for continuous variables and as percentages for categorical variables. For each variable, values before the slash correspond to the medication-exposed group and values after the slash correspond to the matched control group. Variables include demographics, comorbidities, and metabolic parameters relevant to baseline risk stratification across the three exposure cohorts. All matched variables had standardized mean differences (SMD) < 0.1.

* In TriNetX, current age is calculated from birth irrespective of mortality. For atovaquone-proguanil and mefloquine, equivalence of both current age and age at index between arms confirms calendar alignment of index dates.

Abbreviations: SD, standard deviation; BMI, body mass index; HbA1c, hemoglobin A1c; SMD, standardized mean difference.

### 3.6 Analysis C: Cox proportional hazard models

In the TriNetX cohorts, we evaluated mortality and age-related conditions identified in the LHS analyses, excluding individuals with a prior diagnosis of each outcome at baseline. Cumulative incidence plots are presented in Figure 4.

**FIGURE 4:**
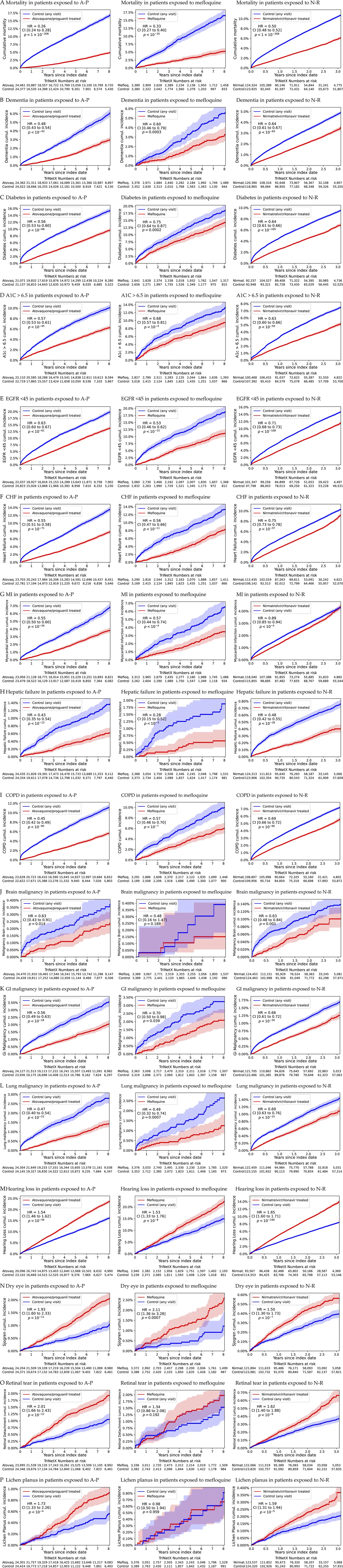
Cumulative incidence of mortality and age-related health outcomes following antiprotozoal exposure in TriNetX cohorts (Analysis C). Cumulative incidence curves comparing patients treated with atovaquone-proguanil (left), mefloquine (middle), and nirmatrelvir-ritonavir (right) versus propensity score-matched controls in the TriNetX network. Outcomes include all-cause mortality and selected age-related conditions identified in the LHS cohort, with follow-up extending up to eight years for atovaquone-proguanil and mefloquine and up to three years for nirmatrelvir-ritonavir. Individuals with prior diagnoses of the respective outcomes at baseline were excluded from each analysis. Hazard ratios (HR), 95% confidence intervals (CI), and p-values were estimated using Cox proportional hazards models. Associations included consistent reductions in mortality and morbidity across multiple outcomes, while increased risks were identified for certain adverse events, including hearing loss and ophthalmic conditions. Abbreviations: CHF, congestive heart failure; CI, confidence interval; COPD, chronic obstructive pulmonary disease; eGFR, estimated glomerular filtration rate; HR, hazard ratio; MI, myocardial infarction; LHS, Leumit Health Services; GI, gastrointestinal. **(A)** mortality; **(B)** dementia; **(C)** diabetes; **(D)** HbA1c threshold outcome; **(E)** eGFR <45; **(F)** congestive heart failure; **(G)** myocardial infarction; **(H)** hepatic failure; **(I)** COPD; **(J)** brain malignancy; **(K)** GI malignancy; **(L)** lung malignancy; **(M)** hearing loss; **(N)** dry eye; **(O)** retinal tear; **(P)** lichen planus.

Consistent and significant reductions in all-cause mortality were observed across all three medications: HR = 0.26 (95% CI, 0.24-0.28; p < 0.001) for A-P, HR = 0.33 (0.27-0.40; p < 0.001) for mefloquine, and HR = 0.50 (0.48-0.52; p < 0.001) for N-R. Significant reductions were found for major chronic diseases. Dementia incidence was reduced (HR = 0.48–0.64 across medications), as were diabetes mellitus diagnoses (HR = 0.56-0.75) and diabetes defined by first occurrence of HbA1c ≥ 6.5% (HR = 0.57-0.68). Kidney disease, defined as an eGFR <45 mL/min/1.73 m^2^, was also significantly reduced (HR = 0.53-0.71). Additional reductions were observed for heart failure (HR = 0.55-0.75), myocardial infarction (HR = 0.55-0.89), hepatic failure (HR = 0.28-0.48), COPD (HR = 0.45-0.69), lung cancer (HR = 0.47-0.69), gastrointestinal malignancies (HR = 0.56-0.70), and brain malignancies (HR = 0.63 for A-P and N-R). Most of the observed associations reached statistical significance (p < 0.05, most <0.001).

As in the LHS cohort, antiprotozoal exposure was associated with higher risks of specific adverse outcomes, including hearing loss (HR = 1.53-1.65), Sjögren’s syndrome/dry eye (HR = 1.50-2.11), retinal tear (HR = 2.01 for A-P and 1.62 for N-R), and lichen planus (HR = 1.73 for A-P and 1.59 for N-R).

In summary, these consistent patterns across independent systems warranted mechanistic and epidemiologic interpretation, addressed in the Discussion.

## 4 Discussion

### 4.1 Principal findings

In this study, we leveraged comprehensive electronic health records from large-scale healthcare networks in two countries to systematically screen for medications associated with increased human lifespan. Using a medication-wide association approach with a 10-year window in a nationwide Israeli cohort, we identified two antiprotozoal agents, atovaquone-proguanil (A-P) and mefloquine, as strongly and significantly associated with reduced all-cause mortality and a broad spectrum of age-related morbidities. In complementary analyses performed using a 5-year window, we also observed consistent associations for nirmatrelvir-ritonavir (N-R), a protease inhibitor with reported antiparasitic activity. These findings were independently validated in the U.S.-based TriNetX network, where consistent reductions in mortality and major age-related outcomes were observed across these medications. Analyses were designed to ensure alignment of time zero between exposed and unexposed groups, thereby preventing immortal time and related time-dependent biases.

This work used an original approach of systematically comparing medication histories over a decade in individuals with above- versus below-average longevity within a large population. Using this approach, we identified a class of medications—antiprotozoals—that were consistently associated with increased lifespan and a reduced incidence of common life-shortening conditions, including diabetes and its complications, cardiovascular and cerebrovascular diseases, chronic kidney disease, hepatic failure, chronic obstructive pulmonary disease, dementia, and various malignancies. Replicating these associations across two independent healthcare systems in different countries, and for three mechanistically related but distinct medications, provides convergent evidence supporting the robustness of these findings. These results raise the possibility that chronic infections, including protozoal pathogens, may contribute to age-related disease risk, although causal inference cannot be established from observational data.

### 4.2 Addressing potential concerns for travel associated bias

A potential concern in interpreting the associations observed for atovaquone-proguanil and mefloquine is confounding related to travel-associated behaviors, including residual differences in baseline health status and healthcare engagement not fully captured by propensity-score matching. The inclusion of nirmatrelvir-ritonavir provides an important complementary context, as this medication is prescribed in a fundamentally different clinical setting. Unlike antimalarial agents used in travelers, nirmatrelvir-ritonavir is administered to individuals at increased risk for adverse outcomes from COVID-19, typically characterized by older age and multiple comorbidities. This context reduces the likelihood that a healthy-user or healthy-traveler effect is the primary explanation for the observed associations. Notably, this exposure context is directionally opposite to that of travel-related prophylaxis, as treatment is preferentially administered to older and multimorbid individuals.

In addition, in our health system, nirmatrelvir-ritonavir was distributed through a centralized, protocol-driven framework based on predefined clinical risk criteria, with provision independent of patient-initiated care. Similar structured eligibility pathways were implemented in other healthcare systems, including those represented in the TriNetX network, where the medication was broadly provided through programmatic allocation (e.g., Medicare and Medicaid coverage). This mode of allocation reduces the influence of disparities related to healthcare access, socioeconomic status, and health-seeking behavior.

The consistency of the findings across independent healthcare systems, distinct exposure contexts, and medications with different primary indications makes simple healthy-user or healthy-traveler explanations less likely. Nevertheless, as with all observational studies, residual confounding cannot be completely excluded and prospective studies will be required to establish causality.

### 4.3 Additional observations and biological signals

Remarkably, we also observed consistent increases in the incidence of certain adverse outcomes, including hearing loss, Sjögren’s syndrome (Negrini et al., 2022), and lichen planus (Le Cleach and Chosidow, 2012), following antiprotozoal exposure. Rather than undermining the protective associations, these findings may offer a plausible, though still speculative, mechanistic explanation that is compatible with the observed associations.

These conditions affect cell types and tissues that are highly sensitive to localized immune-mediated damage, which may arise following the pharmacological elimination of intracellular pathogens. The abrupt clearance of these pathogens could trigger localized inflammation and immune activation at sites of prior intracellular colonization, revealing the pathogen reservoirs while concurrently reducing systemic inflammation.

### 4.4 *Toxoplasma gondii* as a candidate mechanism

We explored several biological explanations that could potentially account for the observed associations. Among possible candidates, *Toxoplasma gondii* is of particular interest because of its high global prevalence, lifelong intracellular persistence, broad tissue tropism, and reported associations with neurological, cardiovascular, metabolic, and malignant diseases. A broader evolutionary and mechanistic framework linking persistent *Toxoplasma gondii* infection with late-life disease and mortality has recently been described in detail (Israel and Israel, 2026). Epidemiologic studies have associated *T. gondii* infection with dementia (Yang et al., 2021), cardiovascular disease (Egorov et al., 2021), schizophrenia (Sutterland et al., 2015), and adverse health outcomes in population-based studies, including recent nationwide matched cohort analyses (Israel S. et al., 2026). Our findings of reduced diabetes and its complications following antiprotozoal treatment are consistent with, though not direct evidence for, the hypothesis that persistent *T. gondii* infection may contribute to systemic inflammation and thromboembolic risk.

*Toxoplasma gondii* is ubiquitous in human environments, with seroprevalence estimates ranging from approximately 20%–80% depending on geography and age. Its intracellular persistence in the gastrointestinal tract and other tissues may promote chronic inflammation and immune evasion. Interference with host-cell apoptosis and immune surveillance has also been proposed as a mechanism potentially linking *T. gondii* to malignancy. In line with this possibility, we previously reported enrichment of *T. gondii* DNA signatures in stool samples from individuals with colorectal cancer, suggestive of active or latent gastrointestinal colonization, although a causal role in malignancy has not been established (Israel et al., 2025).

*Toxoplasma gondii* has also been reported in cutaneous tissues (Leyva and Santa Cruz, 1986), and localized persistence in skin or mucosal sites has been proposed as a potential contributor to chronic inflammation and metabolic dysfunction. Several studies have reported increased *T. gondii* seroprevalence among individuals with diabetes mellitus (Catchpole et al., 2023; Soltani et al., 2021). Systemic dissemination can additionally involve neuronal tissues, and seropositivity has been associated with neuropsychiatric and neurodegenerative disorders, including dementia and schizophrenia (Flegr, 2007; Israel A. et al., 2026; Nayeri et al., 2021; Torrey et al., 2012; Wang J. et al., 2024; Wiener et al., 2020). These observations are consistent with, though not direct evidence for, our finding of reduced dementia incidence following antiprotozoal exposure.

Hematogenous dissemination and associated vascular inflammation have also been proposed as mechanisms linking *T. gondii* infection with cardiovascular and cerebrovascular morbidity (Alhusseiny et al., 2021; Egorov et al., 2021). The reductions in cardiovascular outcomes observed in the present study are compatible with this hypothesis, although alternative explanations remain possible and the present observational data do not establish *T. gondii* as the causal mediator.

### 4.5 Pharmacologic plausibility

The known or reported anti-*Toxoplasma* activity of atovaquone-proguanil, mefloquine, and ritonavir provides pharmacologic plausibility for a protozoal-mediated mechanism. A broader discussion of the potential relevance of these agents to persistent *Toxoplasma gondii* infection and late-life disease has recently been presented (Israel and Israel, 2026).

Atovaquone is a well-established anti-*Toxoplasma* agent that inhibits parasite mitochondrial electron transport and is used in treatment and prophylaxis of toxoplasmosis. Proguanil may enhance its activity through additional effects on mitochondrial and folate metabolism (Spencer and Goa, 1995; KOVACS, 1992). Mefloquine has demonstrated both *in vitro* and *in vivo* activity against *T. gondii*, including reductions in parasite burden in experimental models (Secrieru et al., 2020; El Sharazly et al., 2023). Nirmatrelvir-ritonavir has not been formally studied as a treatment for *T. gondii*; however, ritonavir and related HIV protease inhibitors have demonstrated *in vitro* anti-*Toxoplasma* activity (Alfonso and Monzote, 2011; Derouin and Santillana-Hayat, 2000).

The duration of antimalarial prophylaxis, typically several weeks, together with substantial intracellular exposure, may be sufficient to eliminate or markedly reduce persistent protozoal burden in key reservoirs, providing a plausible mechanism for the durable associations observed after treatment. In a separate matched case-control analysis in LHS, prior mefloquine exposure was less frequent among individuals with positive *T. gondii* serology (OR 0.45, 95% CI 0.27–0.74), providing additional indirect support for an effect on parasite persistence (Israel S. et al., 2026).

Nirmatrelvir-ritonavir provides a distinct exposure context because it is administered for only 5 days, typically to older and multimorbid patients, and, unlike antimalarial prophylaxis, its use is unrelated to travel and therefore does not involve traveler selection. Despite this substantially shorter treatment duration, we observed similar associations with all-cause mortality and age-related morbidities, together with increases in several of the same auditory, visual, and dermatologic outcomes observed with atovaquone-proguanil and mefloquine. These convergent findings motivate direct mechanistic investigation but do not establish parasite eradication as the causal pathway.

### 4.6 Tissue-specific manifestations from clinical signals

The observed associations of antiprotozoal treatment with hearing loss, retinal detachment, Sjögren’s syndrome, and lichen planus are consistent with, and lend indirect support to, the possibility of *T. gondii* involvement, although these tissue-specific signals could also reflect other immune-mediated mechanisms. These outcomes align with the parasite’s known tissue tropism: congenital toxoplasmosis is a leading cause of sensorineural hearing loss in infants (Boyer et al., 2005), seropositivity has been linked to hearing impairment (Getzmann et al., 2024), *Toxoplasma* retinitis is a major cause of posterior uveitis worldwide (Holland, 2003), and cutaneous toxoplasmosis has been described in immunocompromised individuals (Leyva and Santa Cruz, 1986). Taken together, these findings suggest that the observed increases in localized conditions after antiprotozoal therapy may reflect parasite elimination from canonical tissue reservoirs, triggering localized pathology while simultaneously lowering the risk of subsequent systemic spread, although this explanation remains hypothetical.

### 4.7 Interpretation and clinical implications

Until now, the prevailing view has been that *Toxoplasma gondii* is largely innocuous in immunocompetent humans, aside from its teratogenic effects in newly infected pregnant women and its impact on immunocompromised individuals. Consequently, serological testing is rarely performed outside these contexts. In histopathological examinations, the small, easily overlooked cysts characteristic of *T. gondii* are typically disregarded or unreported due to the absence of a recognized clinical correlation. This likely contributes to the under-recognition of its potential long-term harms and to the absence of therapeutic strategies aimed at parasite clearance in the general population.

Based on the epidemiologic observations of this study, we cautiously hypothesize that chronic *T. gondii* colonization could be one of several contributors to aging-related conditions, and that this may partly, though not fully, explain the observed associations between antiprotozoal therapies and reduced mortality and morbidity. These findings should be regarded as hypothesis-generating; they raise, but do not establish, the possibility that antiprotozoal treatment could confer long-term benefits by lowering the burden of *T. gondii*.

### 4.8 Limitations

Despite the strengths of our large-scale, systematically matched analyses and replication across independent populations, this study has several limitations. First, as an observational study, residual confounding cannot be rigorously excluded. However, the consistent observation of both protective and adverse associations (e.g., hearing loss, Sjögren’s disease, lichen planus) across large cohorts from different countries, for two distinct antimalarials, and in TriNetX also for N-R (a medication administered during COVID-19 primarily to older and less healthy patients and provided at no cost across socioeconomic groups) reduces the likelihood that selection or indication bias fully explains the findings.

Second, in the LHS exploratory analysis (Analysis A), individuals exposed to antimalarials did exhibit slightly healthier baseline characteristics, which may explain part, though not all, of the observed risk reduction. In this design, survival status was used to define cases and controls, reflecting its role as an exploratory, hypothesis-generating screen rather than a causal inference framework. While it could in principle amplify baseline differences between groups, these concerns were addressed in Analyses B and C, where group assignment was based directly on first medication exposure (defining the index date) and outcomes were analyzed longitudinally using survival models. In particular, Analysis C further minimized bias through propensity-score matching, which ensured rigorous baseline balance across demographic and clinical factors, including race/ethnicity, BMI, diabetes status, HbA1c, and smoking status. Multiple outcomes were evaluated across several cohorts. Although correction for multiple testing was applied during screening analyses, not all downstream outcome analyses were adjusted for multiplicity. Consequently, some associations may represent false-positive findings and should be interpreted in the context of replication across independent cohorts.

Finally, the replication across two independent healthcare systems supports the interpretation that these signals are unlikely to be artifacts of a single study design. The persistence of associations with age-related diseases of distinct etiology and disease course, and across outcomes that often occur in otherwise healthy individuals (such as malignancy and myocardial infarction), further reduces the likelihood of confounding as the primary explanation. Moreover, having constructed the cohorts appropriately and empirically assessed and reduced the risk of major biases through baseline comparisons, observing concordant protective effects together with specific adverse associations (hearing loss, Sjögren’s disease, lichen planus) across drugs with unrelated primary indications provides additional support that the findings are unlikely to be primarily attributable to indication bias. Nevertheless, one cannot exclude that the magnitude of observed associations, particularly for mortality, may reflect a combination of causal and non-causal effects and should be interpreted accordingly. The magnitude of several observed associations, particularly for mortality, is larger than typically observed following short-duration pharmacologic exposures. While replicated across cohorts and drugs, these findings should be interpreted cautiously because residual confounding, selection mechanisms, and unmeasured differences between exposed and unexposed individuals may contribute to the observed effect sizes. Alternative explanations include microbiome-mediated effects independent of protozoal eradication, immunomodulatory properties of the studied medications, differences in healthcare engagement, and residual confounding despite matching procedures. Generalizability to low- and middle-income countries remains uncertain, as protozoal exposure prevalence, healthcare access, medication availability, prescribing practices, and competing causes of morbidity differ substantially from those observed in Israel and the United States.

## 5 Conclusion

Overall, the observed protective effects of antiprotozoal medications against mortality and a broad range of aging-associated diseases—together with consistent increases in conditions anatomically linked to canonical reservoirs of *Toxoplasma gondii*, observed across two countries and three drugs with distinct indications—generate a hypothesis, to be tested in future studies, that chronic protozoal colonization, possibly involving *T. gondii* among other mechanisms, may be an underrecognized and currently unproven contributor to age-related morbidity. The spectrum of affected outcomes, spanning metabolic, cardiovascular, cerebrovascular, neurodegenerative, and malignant diseases, suggests that, if confirmed, this hypothesis could have broader public health relevance. These results raise the possibility that targeted eradication or suppression of protozoal infections could represent a potential avenue for future investigation in reducing age-related disease burden. This hypothesis can be evaluated in future studies, including serologic or microbiome-based approaches. Mechanistic studies, serologic investigations, microbiome analyses, and prospective randomized trials will be necessary to determine whether the observed associations reflect causal effects.

## Data Availability

The datasets presented in this article are not readily available because the LHS patient-level data generated and analyzed in this study are not publicly available due to patient privacy regulations. Access may be granted to qualified researchers subject to approval by the Leumit Health Services Institutional Review Board. TriNetX data are accessible only to licensed member organizations of the TriNetX network, in compliance with HIPAA and institutional guidelines. Requests to access the datasets should be directed to AI, arielisrael@tauex.tau.ac.il.
