# Supplementary material for: Antiprotozoal drug exposure and reduced long-term mortality and morbidity: a multi-cohort pharmacoepidemiologic study
Source: Front Pharmacol. 2026 Oct 27;17:1861376. doi: 10.3389/fphar.2026.1861376 (PMC13748354; doi:10.3389/fphar.2026.1861376)
Supplement: Supplementary file 1 [file Table1.docx]

## Supplementary Table 1. Comparison of medication purchases in the 10 years preceding the index date in the case (shorter lifespan) and control (longer lifespan) groups of the LHS cohort

| **Medication** | **Case**  **N=14,986** | **Control**  **N=14,986** | **Odds Ratio**  **[95% CI]** | **p-value** | **FDR** |
| --- | --- | --- | --- | --- | --- |
| Mefloquine | 11 (0.07%) | 34 (0.23%) | 0.32 [0.15-0.65] | 0.00082 | 0.0001 |
| Atovaquone + Proguanil | 27 (0.18%) | 62 (0.41%) | 0.43 [0.27-0.69] | 0.00026 | 0.0002 |

Counts represent individuals with at least one recorded purchase of the medication in the 10 years preceding the index date. Odds ratios (OR) with 95% confidence intervals (CI) were estimated using Fisher’s exact test, with p-values corrected for multiple testing using the Benjamini–Hochberg false discovery rate (FDR) procedure. The screen evaluated 1,470 distinct compounds; among the highest-ranking associations, the antiprotozoal ATC class P01B (antimalarials) was the only therapeutic category represented by multiple compounds within the top entries, which motivated its selection for downstream validation.

## Supplementary Table 2. Medications meeting the pre-specified screening criteria in the exploratory matched case-control longevity analysis

| ATC | **ATC label** | **OR (CI)** | **pval** | **FDR BH** |
| --- | --- | --- | --- | --- |
| G03CCWW PO | Estrogens, Combinations With Other Drugs PO | 0.32 [0.11 to 0.77] | 0.0081 | 0.020032 |
| P01BC02 PO | Mefloquine PO | 0.32 [0.15 to 0.65] | 0.000817 | 0.002395 |
| G03FA01 EX | Norethisterone and E EX | 0.41 [0.23 to 0.70] | 0.000527 | 0.00158 |
| D03AX03 II | Dexpanthenol II | 0.42 [0.22 to 0.77] | 0.003807 | 0.010059 |
| P01BB51 PO | proguanil and atovaq PO | 0.42 [0.23 to 0.77] | 0.003173 | 0.00846 |
| C05AD01 # | Lidocaine # | 0.43 [0.20 to 0.87] | 0.016518 | 0.037973 |
| P01BX51 PO | Atovaquone/proguanil PO | 0.43 [0.20 to 0.87] | 0.016518 | 0.037973 |

This table presents all medication exposures that met the pre-specified screening criteria in Analysis A, the exploratory matched case-control screening analysis comparing individuals with exceptional longevity and matched controls. Odds ratios (ORs), 95% confidence intervals (CIs), nominal p-values, and Benjamini-Hochberg false discovery rate (FDR) adjusted p-values are shown. These findings should be interpreted as hypothesis-generating signals identified during the screening phase and were used to guide subsequent analyses. The screening analysis was not designed to establish causal relationships, and exposures not pursued in downstream analyses should not be interpreted as validated longevity-associated factors.

Note: Atovaquone/proguanil was represented by two distinct ATC codes within the Leumit Health Services database (P01BB51 and P01BX51). Both codes were identified independently during the screening analysis and yielded highly similar effect estimates, providing internal consistency for the observed association.

Supplementary Table 3. Exploratory matched comparison of nirmatrelvir–ritonavir recipients and matched controls in Leumit Health Services

| ATC | **ATC label** | **OR (CI)** | **pval** | **FDR BH** |
| --- | --- | --- | --- | --- |
| J05AE30 PO | **nirmatrelvir and ritonavir PO** | 0.28 [0.23 to 0.33] | <0.001 | 2E-70 |

This table presents the results of an exploratory matched comparison between nirmatrelvir–ritonavir recipients and matched controls performed following identification of nirmatrelvir–ritonavir in the secondary five-year screening analysis, to provide additional context regarding the nirmatrelvir–ritonavir signal identified during in this analysis. As with all exploratory analyses, the results should be interpreted cautiously and should not be considered evidence of a causal effect.
